# An important role of the pepper phenylalanine ammonia-lyase gene (*PAL1*) in salicylic acid-dependent signalling of the defence response to microbial pathogens

**DOI:** 10.1093/jxb/eru109

**Published:** 2014-03-18

**Authors:** Dae Sung Kim, Byung Kook Hwang

**Affiliations:** Laboratory of Molecular Plant Pathology, College of Life Sciences and Biotechnology, Korea University, Anam-dong, Sungbuk-ku, Seoul 136-713, Republic of Korea

**Keywords:** *Arabidopsis*, defence, pepper, phenylalanine ammonia-lyase, *Xanthomonas campestris* pv. *Vesicatoria*.

## Abstract

Phenylalanine ammonia-lyase (PAL) has a crucial role in secondary phenylpropanoid metabolism and is one of the most extensively studied enzymes with respect to plant responses to biotic and abiotic stress. Here, we identified the pepper (*Capsicum annuum*) *PAL* (*CaPAL1*) gene, which was induced in pepper leaves by avirulent *Xanthomonas campestris* pv. *vesicatoria* (*Xcv*) infection. *CaPAL1*-silenced pepper plants exhibited increased susceptibility to virulent and avirulent *Xcv* infection. Reactive oxygen species (ROS), hypersensitive cell death, expression of the salicylic acid (SA)-dependent marker gene *CaPR1*, SA accumulation, and induction of PAL activity were significantly compromised in the *CaPAL1*-silenced pepper plants during *Xcv* infection. Overexpression (OX) of *CaPAL1* in *Arabidopsis* conferred increased resistance to *Pseudomonas syringae* pv. *tomato* (*Pst*) and *Hyaloperonospora arabidopsidis* infection. *CaPAL1*-OX leaves exhibited restricted *Pst* growth, increased ROS burst and cell death, and induction of *PR1* expression and SA accumulation. The increase in PAL activity in healthy and *Pst-*infected leaves was higher in *CaPAL1*-OX plants than in wild-type *Arabidopsis.* Taken together, these results suggest that CaPAL1 acts as a positive regulator of SA-dependent defence signalling to combat microbial pathogens via its enzymatic activity in the phenylpropanoid pathway.

## Summary

Phenylalanine ammonia-lyase (PAL) is an inducible enzyme that responds to biotic and abiotic stresses. Results suggest the potential significance of pepper PAL1 in the plant defence response to microbial pathogens.

## Introduction

Plants have evolved multiple defence signalling pathways to cope with adverse environmental conditions and pathogen attack (Jones and Dangl, 2006). The levels of secondary metabolites such as phenylpropanoids are controlled in response to environmental cues (Dixon and Paiva, 1995; Payyavula *et al.*, 2012). The evolutionary emergence of the phenylpropanoid pathway in plants is an important adaptation that enables plant defence against abiotic and biotic stresses (Ferrer *et al.*, 2008). Phenylpropanoid compounds are precursors to a wide range of phenolic compounds, such as flavonoids, isoflavonoids, anthocyanins, plant hormones, phytoalexins, and lignins (Dixon and Paiva, 1995; La Camera, *et al.*, 2004). Phenylpropanoids have important functions in several different pathways: in plant defence against pathogens and predators, in protection from UV irradiation, in signal transduction and communication with other organisms, and as regulatory molecules (Hahlbrock and Scheel, 1989; Dixon and Paiva, 1995; Ferrer *et al.*, 2008; Vogt, 2010).

Phenylpropanoids are derived from cinnamic acid, which is formed from phenylalanine (Vogt, 2010). Phenylalanine ammonia-lyase (PAL) catalyses the non-oxidative deamination of phenylalanine to *trans*-cinnamate. This is the first step in the phenylpropanoid pathway, and is an important regulation point between primary and secondary metabolism (Hahlbrock and Scheel, 1989; Dixon and Paiva, 1995; Huang *et al.*, 2010; Vogt, 2010). PAL is an inducible enzyme that responds to biotic and abiotic stresses such as pathogens, UV irradiation, and low temperature (Dixon and Paiva, 1995; MacDonald and D’Cunha, 2007). PAL plays an important role in plant defence; it is involved in the biosynthesis of salicylic acid (SA), an essential signal involved in plant systemic resistance (Mauch-Mani and Slusarenko, 1996; Nugroho *et al.*, 2002; Chaman *et al.*, 2003). *PAL* gene expression responds to a variety of environmental stresses, including pathogen infection, wounding, nutrient depletion, UV irradiation, and extreme temperatures (Edwards *et al.*, 1985; Liang *et al*., 1989a, b; Huang *et al.*, 2010; Payyavula *et al.*, 2012; Jin *et al.*, 2013).

Molecular genetics methods have been used to silence or disrupt *PAL* genes for the functional analysis of their roles in plant development and responses to external stimuli (Pallas *et al.*, 1996; Rohde *et al.*, 2004; Huang *et al.*, 2010). In *Arabidopsis thaliana*, PAL is encoded by a small gene family with four members, denoted *PAL1−PAL4* (Raes *et al.*, 2003; Huang *et al.*, 2010). *PAL1*, *PAL2*, and *PAL4* are strongly expressed in inflorescent stems, a tissue rich in lignifying cells, whereas the *PAL3* transcript is expressed at a very low level (Raes *et al.*, 2003). Phenotypic analysis of the *PAL1* and *PAL2* genes was performed for both single and double mutants. The growth and development phenotypes of the *pal1* and *pal2* single mutants were not significantly different from the growth and development phenotypes of wild-type (WT) plants. The *pal1/pal2* double mutant did show phenotypic differences from that of WT plants, such as infertility, a significant reduction in lignin accumulation, and alteration in secondary cell-wall ultrastructure (Rohde *et al.*, 2004). Recently, morphology-based genetic analysis has been conducted on *pal1/pal2* double mutants and *pal1/pal2/pal3/pal4* quadruple mutants. The *pal1/pal2* double mutant produced slightly yellow seeds due to the lack of condensed tannin pigments in the seed coat, it was deficient in anthocyanin pigments in various plant tissues, and it was highly sensitive to UV-B light. These results suggest that PAL1 and PAL2 have important and redundant roles in flavonoid biosynthesis (Huang *et al.*, 2010). The *pal1/pal2/pal3/pal4* quadruple mutant had a stunted phenotype, substantially reduced levels of SA accumulation, and increased susceptibility to the virulent bacterial pathogen *Pseudomonas syringae* (Huang *et al.*, 2010).

*PAL*-suppressed transgenic tobacco has undeveloped systemic acquired resistance, which correlates with the reduction of SA levels in both inoculated and uninoculated leaves (Pallas *et al.*, 1996). The PAL inhibitor 2-aminoindan-2-phosphonic acid decreases the pathogen- or elicitor-induced SA accumulation in potato (*Solanum tuberosum*), cucumber (*Cucumis sativus*), and *Arabidopsis* (Meuwly *et al.*, 1995; Mauch-Mani and Slusarenko, 1996; Coquoz *et al.*, 1998). Mauch-Mani and Slusarenko (1996) proposed that the production of SA precursors is a major function of PAL in the resistance of *Arabidopsis* to *Hyaloperonospora arabidopsidis* (*Hpa*).

In this study, we identified the pepper (*Capsicum annuum*) *PAL* gene, *CaPAL1*, using the macroarray method (Jung and Hwang, 2000). *CaPAL1* was upregulated in pepper leaves by infection with avirulent *Xanthomonas campestris* pv. *vesicatoria* (Xcv) Bv5-4a. *CaPAL1* loss of function and gain of function were characterized using virus-induced gene silencing (VIGS) in pepper plants (Choi *et al.*, 2007) and ectopic overexpression in *Arabidopsis* plants (Kim and Hwang, 2011). *CaPAL1-*silenced pepper plants exhibited increased susceptibility to *Xcv* infection. *CaPAL1* silencing promoted *Xcv* growth but suppressed pathogen-induced PAL activity in pepper leaves. Suppression of *CaPAL1* induction in silenced leaves reduced the expression of the SA-dependent gene *CaPR1*, but did not reduce the expression of the jasmonic acid-dependent gene *CaDEF1* (defensin). *CaPAL1* silencing also compromised the SA accumulation, oxidative burst, and cell death during *Xcv* infection. Constitutive overexpression of *CaPAL1* in *Arabidopsis* conferred increased PAL activity, increased reactive oxygen species (ROS) burst and cell death, induction of *PR1* expression and SA accumulation, reduced susceptibility to *Pseudomonas syringae* pv*. tomato* (*Pst*), and increased basal resistance to infection by the obligate biotrophic oomycete *Hpa*. Together, these results provide convincing evidence for the involvement of pepper *CaPAL1* in plant defence against pathogen attack. This suggests the potential significance of *PAL* genes in plant immune responses.

## Materials and methods

### Plant materials and growth conditions

Pepper (*C. annum* L., cv. Nockwang) plants were grown in plastic pots containing a soil mix (peat moss:perlite:vermiculite, 2:1:1, v/v/v) in a growth room at 28 °C with a day length of 16h and a light intensity of 70 μmol photons m^–2^ s^–1^. All *A. thaliana* lines used in this study were the ecotype Columbia (Col-0). Seeds were surface sterilized and cold treated for 3 d at 4 °C before they were sown in the soil mix. Plants were grown in the soil mix in an environmental growth chamber at 24 °C under 14h light/10h dark cycles with a photosynthetic flux of 130 μmol photons m^–2^ s^–1^ and 60% relative humidity.

### Pathogen inoculation

*Xcv* strains Ds1 and Bv5-4a, which are virulent and avirulent to pepper plants (cv. Nockwang), respectively, were used in this study. The bacteria were cultured in yeast nutrient (YN) broth (5g l^–1^ of yeast extract and 8g l^–1^ of nutrient broth), harvested by centrifugation at 10000*g* for 2min, and resuspended in sterilized tap water. To prepare the inoculum for inoculation, the bacterial suspension was diluted to an appropriate density. Leaves of pepper plants at the six-leaf stage were inoculated by infiltrating the bacterial suspension using a syringe without a needle (Choi and Hwang, 2011).

*Pst* DC3000 and DC3000 (*avrRpm1*), which are virulent and avirulent to *Arabidopsis* Col-0, respectively, were grown in King’s B broth (10g l^–1^ of peptone, 1.5g l^–1^ of K_2_HPO_4_, 15g l^–1^ of glycerol, and 5g l^–1^ of MgSO_4_). Leaves of *Arabidopsis* plants were infiltrated with *Pst* in 10mM MgCl_2_ using a syringe without a needle.

*Hpa* isolate Noco2, which is virulent to *Arabidopsis* ecotype Col-0, was used in this study. Spore suspension (5×10^4^ conidiospores ml^–1^) was sprayed onto 7-d-old seedlings. Inoculated plants were domed with a plastic wrap to maintain moisture. The numbers of sporangiophores and spores on cotyledons were counted to assess disease severity at 7 d after inoculation.

### Isolation and sequence analysis of *CaPAL1* cDNA

A cDNA library was constructed from poly(A)^+^ mRNA of pepper leaves inoculated with the incompatible *Xcv* Bv5-4a strain. Pathogen-inducible cDNAs were screened using the macroarray method of Jung and Hwang (2000). Among the screened defence response genes, the PAL homologue, *CaPAL1* (GenBank accession no. KF279696), was selected based on the BlastX algorithm of the NCBI website (Altschul *et al.*, 1997). Amino acid sequence alignment of CaPAL1 and other PAL orthologues was performed using Clustal X (Thompson *et al.*, 1997).

### Genomic DNA gel blot analysis

For Southern blot analysis, genomic DNA was extracted from pepper leaves using Plant DNAzol^®^ Reagent (Invitrogen, Carlsbad, CA) following the manufacturer’s instructions. Twenty micrograms of genomic DNA was digested with restriction endonucleases *Eco*RI and *Hin*dIII, subjected to electrophoresis through 0.7% agarose gels, and blotted onto Hybond^TM^-N+ membranes (Amersham, Little Chalfont, UK), followed by cross-linking under UV illumination. *CaPAL1* cDNA probes were [^32^P]dCTP labelled using Klenow enzyme (Roche, Mannheim, Germany). The membranes were hybridized to ^32^P-labelled probe at 65 °C overnight (Jung *et al.*, 2003).

### RNA gel blot and real-time reverse transcription (RT)-PCR analyses

Total RNA was extracted from pepper and *Arabidopsis* plants with Trizol reagent (Invitrogen) according to the manufacturer’s instructions. To generate the *CaPAL1* gene-specific probe for the northern blot, the full-length *CaPAL1* cDNA was [^32^P]dCTP-labelled using Klenow enzyme. RNA gel blot and real-time RT-PCR analyses were conducted following standard procedures, as described previously (Choi and Hwang, 2011; Choi *et al.*, 2012).

For real-time RT-PCR analysis, cDNA was synthesized from 1 μg of total RNA using avian myeloblastosis virus reverse transcriptase (Roche), synthesized oligo(dT)_15_ primer, and dNTPs (Takara, Shiga, Japan). The real-time PCR was performed for 45 cycles using 1 μl of cDNA as template. The PCR conditions were 94 °C for 5min; 30 cycles of 94 °C for 30 s, 55 °C for 30 s, and 72 °C for 90 s; and 72 °C for 10min for the final extension. The primers used for RT-PCR were 5′-GGTTTTGGTGCAACATCACATAGGAG-3′ (forward) and 5′-ATTGTCAAAGTTCTCTTAGCTACTTGGC-3′ (reverse) for *CaPAL1*, 5′-CATCAGGAAGGACTTGTACGG-3′ (forward) and 5′-GATGGACCTGACTCGTCATAC-3′ (reverse) for *ACT1* in *Arabidopsis*, 5′-CAGGATGCAACACTCTGGTGG-3′ (forward) and 5′-ATCAAAGGCCGGTTGGTC-3′ (reverse) for *CaPR1*, 5′-CAAGGGAGTATGTGCTAGTGAGAC-3′ (forward) and 5′-TGCACAGCACTATCATTGCATAC-3′ (reverse) for *CaDEF1*, and 5′-AAACGGCTACCACATCCAAG-3′ (forward) and 5′-ACCCATCCCAAGGTTCAACT-3′ (reverse) for the 18S rRNA gene in pepper.

### VIGS

pTRV (tobacco rattle virus)-based VIGS was conducted to generate *CaPAL1* knockdown (TRV:*CaPAL1*) pepper plants (Liu *et al.*, 2002; Choi *et al.*, 2007). The N-terminal fragment of *CaPAL1* cDNA (5′ 546bp) was inserted into pTRV2. pTRV1 and pTRV2:*00* or pTRV2:*CaPAL1* in *Agrobacterium tumefaciens* GV3101 were co-infiltrated into the fully expanded cotyledons of pepper plants. Gene knockdown efficacy in *CaPAL1*-silenced pepper plants was confirmed by RT-PCR from the leaves infected with *Xcv*.

### Plant transformation

The cDNA fragment of *CaPAL1* was cloned into the pBIN35S plant binary vector. The primer pairs 5′-GAGATCTAGAATGG CATCAACAATTGC-3′ (forward) and 5′-GAGAGGATCCCGCT TCCAAGATCTCAA-3′ (reverse) were used in the PCR to create *Xba*I- and *Bam*HI*-*sited *CaPAL1* cDNA. The resulting binary plasmid was transformed into *A. tumefaciens* strain GV3101 by electroporation. *Agrobacterium*-mediated transformation of *Arabidopsis* ecotype Col-0 was performed using the floral dipping method of Clough and Bent (1998). Transformants were selected on 0.5× Murashige and Skoog (MS) agar plates containing 50 μg ml^–1^ of kanamycin. Successful transformation was confirmed by RT-PCR using the *CaPAL1* gene-specific primer described above. Among the selected transgenic plants, lines #2, #3, and #7 were used in this study.

### PAL activity assay

Total proteins were extracted from pepper and *Arabidopsis* leaves using 100mM phosphate buffer (pH 6.0) containing 2mM EDTA, 4mM dithiothreitol, and 2% (w/w) polyvinylpyrrolidone. PAL activity in the leaf extract was determined by the method of Song and Wang (2011), with slight modifications. Briefly, the protein extract (0.2ml) was incubated at 30 °C for 60min with 2ml of 0.01M borate buffer (pH 8.7) and 1ml of 0.02M l-phenylalanine (pre-dissolved in 0.01M borate buffer pH 8.7). Absorbance was measured at 290nm before and after incubation. One unit of activity (katal) was defined as the amount of PAL that produces 1 mole of cinnamic acid in 1s and was expressed as nkatal mg^–1^ of protein. A reaction without the substrate was used as a blank control. Triplicate assays were performed for each extract.

### Measurement of H_2_O_2_ and ion conductivity

H_2_O_2_ levels in pepper leaves were quantified using a xylenol orange assay (Gay *et al.*, 1999; Choi *et al.*, 2007). The xylenol orange assay reagent was freshly prepared. Two hundred microliters of solution [25mM FeSO_4_ and 25mM (NH_4_)_2_SO_4_ in 2.5M H_2_SO_4_] was added to 20ml of 125 μM xylenol orange in 100mM sorbitol. Eight leaf discs (0.4cm in diameter) were excised from pepper leaves and floated on 1ml of distilled water for 1h. One hundred microliters of the supernatant was immediately added to 1ml of xylenol orange assay reagent. The reaction mixture was incubated for 30min at room temperature, and H_2_O_2_ production was monitored by measuring the absorbance at 560nm using a DU 650 spectrophotometer (Beckman, Urbana, IL, USA).

Ion conductivity was measured using a SensION7 conductivity meter (Hach, Loveland, CO, USA) to quantify cell death in pepper leaves. Leaf discs (1.2cm in diameter) were excised from pepper leaves inoculated with *Xcv* and washed for 30min in 20ml of distilled water. Ion conductivity was monitored after incubation of the leaf discs for 3h in freshly prepared distilled water (20ml).

### SA measurement

SA and SA glycoside were extracted and measured from pepper and *Arabidopsis* leaves, as described previously (Aboul-Soud *et al.*, 2004; Kim and Hwang, 2011). Leaf tissues (0.5g) were extracted in 1ml of 90% methanol following homogenization in liquid nitrogen. 3-Hydroxybenzoic acid (Sigma) was used as an internal standard. SA extracts were analysed automatically through a fluorescence detector (excitation at 305nm and emission at 405nm) in reversed-phase high-performance liquid chromatography in a Waters 515 system (Waters, Milford, MA, USA) with a C18 column.

## Results

### Isolation and sequence analyses of *CaPAL1*

To isolate pathogen-inducible genes from the pepper cDNA library, differential hybridization was performed based on a macroarray method (Jung and Hwang, 2000). Among the isolated clones, a full-length cDNA encoding a PAL homologue in pepper plants was selected and designated *CaPAL1* based on the BlastX algorithm of the NCBI website (Altschul *et al.*, 1997). The full-length *CaPAL1* cDNA is 2318bp, and contains a 70bp 5′ untranslated region, a 2154bp coding region that encodes a protein of 717 aa, and a 94bp 3′ untranslated region (Supplementary Fig. S1A at *JXB* online). The deduced amino acid sequence of the *CaPAL1* cDNA sequence was compared with plant PAL proteins of tomato, potato, tobacco, *Ipomoea*, and *Arabidopsis*. The sequence identities ranged from 82 to 93%. The PAL domain was placed at aa 199−215 in the *CaPAL1* sequence (Supplementary Fig. S1B). The strict conservation of a PAL domain suggests that CaPAL1 may act as a PAL enzyme in nitrogen metabolism, phenylpropanoid biosynthesis, and alkaloid biosynthesis in pepper plants (Fritz *et al.*, 2006).

To determine how many *PAL* genes are in pepper, pepper genomic DNA was digested with restriction enzymes *Eco*RI and *Hin*dIII, which do not have the recognition sites within the *CaPAL1* cDNA. Hybridization of the *Eco*RI and *Hin*dIII genome digests with the full-length *CaPAL1* cDNA showed three and four bands in the range of 2.7–7.1kb, respectively (Supplementary Fig. S2 at *JXB* online). The full-length *CaPAL1* cDNA (2154bp) was used as a positive hybridization control. These results indicated that the *CaPAL* gene family is composed of three to four genes in pepper plants.

### *CaPAL1* is induced by avirulent *Xcv* infection

Expression of the *CaPAL1* gene in different pepper organs was examined by RNA gel blot analysis. *CaPAL1* transcript levels were barely detectable in stem and flower tissues, but expression was constitutively much higher in roots. Healthy leaf and fruit tissues did not constitutively express *CaPAL1* (Supplementary Fig. S3A at *JXB* online). We investigated whether *CaPAL1* is induced in pepper leaves by virulent or avirulent *Xcv* infection (Supplementary Fig. S3B). Infection with the avirulent *Xcv* Bv5-4a (incompatible interaction), but not with the virulent *Xcv* Ds1, strongly induced *CaPAL1* in pepper leaves. *CaPAL1* induction remained extremely high 5–25h after avirulent *Xcv* Bv5-4a infection. *CaPAL1* transcripts were not detected in mock-inoculated leaves. The virulent *Xcv* Ds1 infection did not effectively induce *CaPAL1* in pepper leaves compared with the induction in response to avirulent *Xcv* Bv5-4a infection.

### *CaPAL1* silencing in pepper confers increased susceptibility to *Xcv* infection

To investigate loss of function of *CaPAL1* in pepper plants, we silenced *CaPAL1* in pepper plants using the tobacco rattle virus (TRV) VIGS technique (Liu *et al.*, 2002; Chung *et al.*, 2004; Kim and Hwang, 2011). To increase the specificity of silencing, the 5′ partial sequence (546bp) of the *CaPAL1* coding region was used to construct pTRV2:*CaPAL1*.

**Fig. 1.**
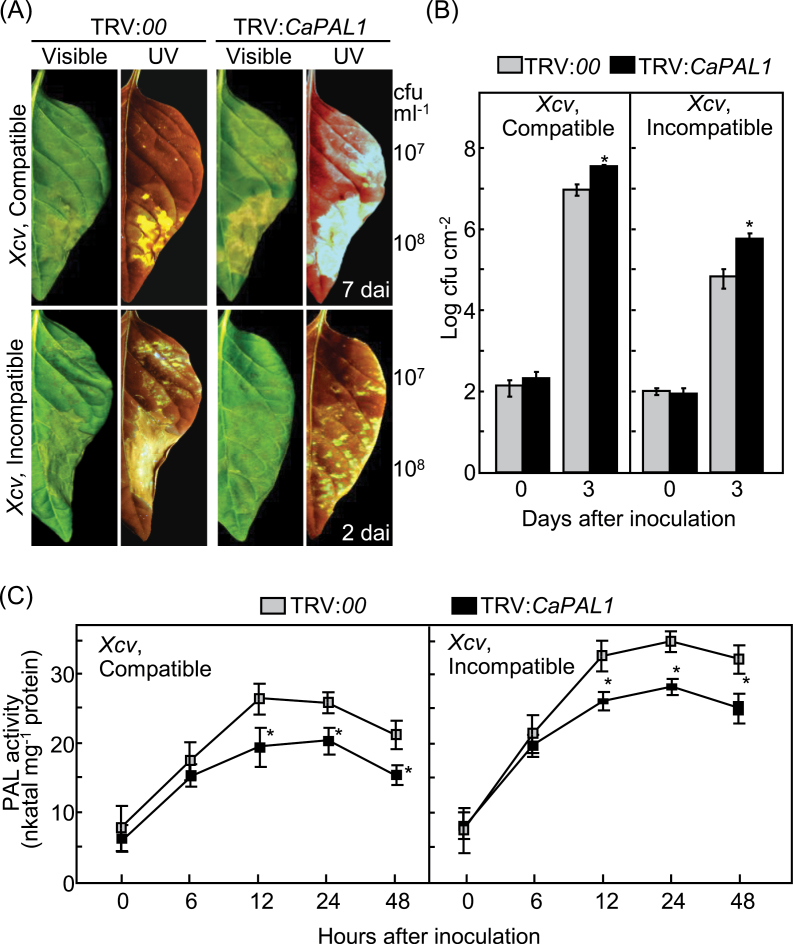
Increased susceptibility of *CaPAL1*-silenced pepper plants to infection by *Xcv*. (A) Disease symptoms developed on empty-vector control (TRV:*00*) and silenced (TRV:*CaPAL1*) leaves 7 and 3 d after inoculation with virulent and avirulent *Xcv* (10^7^ and 10^8^ cfu ml^–1^), respectively. (B) Bacterial growth in empty-vector control and silenced leaves infected with *Xcv* (5×10^4^ cfu ml^–1^). (C) PAL activity in empty-vector control and silenced leaves infected with *Xcv* (10^7^ cfu ml^–1^). In (B) and (C), data are the means±standard deviation (SD) from three independent experiments. Asterisks indicate significant differences, as determined by Student’s *t*-test (*P<0.05). (This figure is available in colour at *JXB* online.)

Silencing of *CaPAL1* in pepper plants resulted in a susceptible response to *Xcv* infection (Fig. 1). The disease phenotypes of *CaPAL1*-silenced plants were compared with those of empty-vector control plants after inoculation with virulent and avirulent *Xcv* at 10^7^ and 10^8^ colony-forming units (cfu) ml^–1^, respectively. *CaPAL1*-silenced pepper leaves exhibited more severe chlorotic lesions 7 d after virulent *Xcv* inoculation compared with that of the empty-vector control. The susceptible regions were intensely fluorescent under UV illumination, indicating the increased accumulation of fluorescent phenolic compounds in silenced leaves (Fig. 1A). Localized hypersensitive response (HR)-like cell death was initiated in the empty-vector control leaves 2 d after inoculation with 10^7^ cfu ml^–1^ of avirulent strain Bv5-4a; however, the HR-like cell death response significantly decreased in *CaPAL1*-silenced leaves during infection. Under UV illumination, dark yellow fluorescent lesions (characteristic of HR) were more numerous in the empty-vector control leaves than those in *CaPAL1*-silenced leaves (Fig. 1A). These disease phenotypes correlated with the proliferation of *Xcv* in silenced leaves. *Xcv* growth in *CaPAL1*-silenced leaves was significantly higher than that in empty-vector control leaves 3 d after inoculation with 5×10^4^ cfu ml^–1^ virulent and avirulent *Xcv* strains (Fig. 1B).

We next investigated whether *CaPAL1* silencing affected PAL activity in pepper leaves during *Xcv* infection (Fig. 1C). Avirulent (incompatible, Bv5-4a) *Xcv* infection induced significantly higher PAL activity than did virulent (compatible, Ds1) *Xcv* infection in both the empty-vector control and *CaPAL1*-silenced leaves. The induction of PAL activity by *Xcv* infection was distinctly compromised in *CaPAL1*-silenced leaves compared with that in empty-vector control leaves (Fig. 1C). These results indicated that *CaPAL1* silencing promotes *Xcv* growth and suppresses pathogen-induced PAL activity in pepper leaves.

### *CaPAL1* silencing compromises the oxidative burst and cell death during *Xcv* infection

**Fig. 2.**
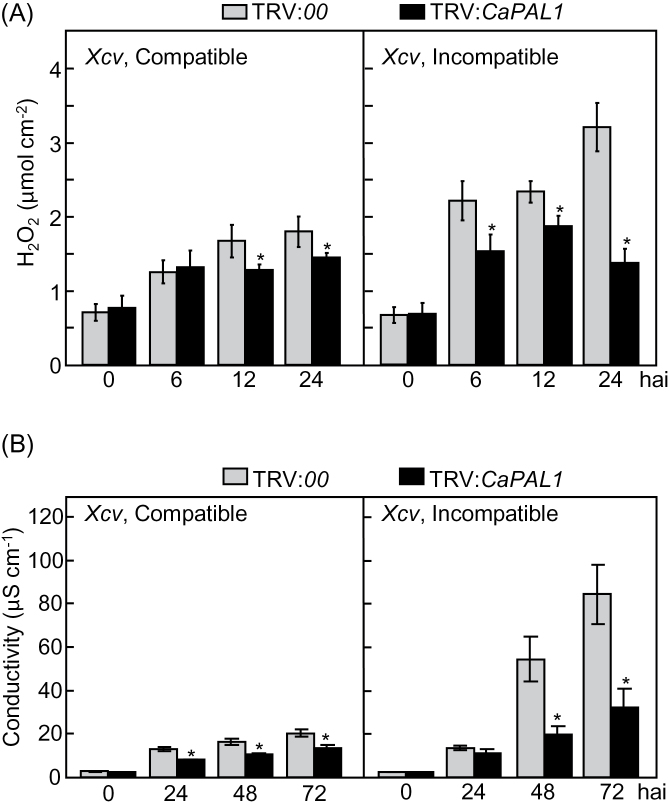
Reduced ROS burst and cell death response in leaves of *CaPAL1*-silenced pepper plants infected with *Xcv*. (A) Quantification of H_2_O_2_ at different time points after inoculation with *Xcv* (10^7^ cfu ml^–1^). (B) Quantification of electrolyte leakage from leaves at different time points after inoculation with *Xcv* (10^7^ cfu ml^–1^). Data are the means±SD from three independent experiments. Asterisks indicate significant differences, as determined by Student’s *t*-test (*P<0.05).

We analysed ROS and the HR cell death in empty-vector control and *CaPAL1*-silenced leaves during *Xcv* infection (Fig. 2). Silencing of *CaPAL1* significantly attenuated the production of H_2_O_2_ by virulent or avirulent *Xcv* infection (Fig. 2A). The visual scores of the cell death phenotype were substantiated by an electrolyte leakage assay. The level of ion (electrolyte) leakage was significantly lower in *CaPAL1*-silenced leaves than that in empty-vector control leaves during virulent *Xcv* infection. Notably, the induction of ion leakage drastically declined in *CaPAL1-*silenced pepper leaves 48h after the avirulent *Xcv* infection (Fig. 2B). Taken together, virulent *Xcv* infection did not significantly induce different phenotypes with respect to H_2_O_2_ production and cell death; however, significantly reduced H_2_O_2_ production and cell death were observed in the *CaPAL1*-silenced leaves during avirulent *Xcv* infection.

### VIGS of *CaPAL1* alters pepper defence-related gene expression and SA accumulation

**Fig. 3.**
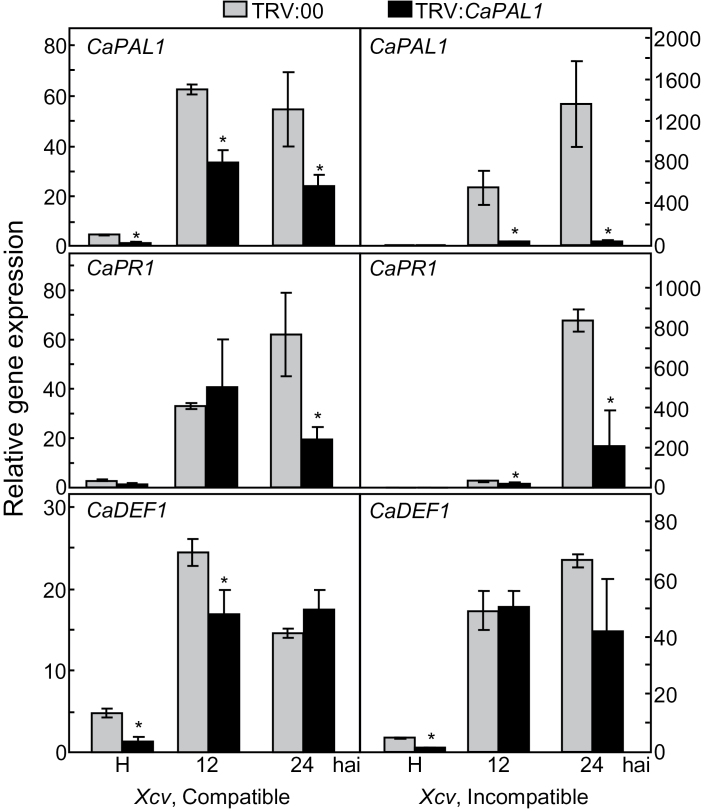
Real-time RT-PCR analysis of *CaPAL1* expression and defence-related genes in empty-vector control and *CaPAL1*-silenced leaves infected with *Xcv*. H, healthy leaves; hai, hours after infection; *CaPR1*, pathogenesis-related protein; *CaDEF1*, defensin. The *C. annuum* 18S RNA gene was used as an internal control. The control sample was normalized to 1. Data are the means±SD (*n*=3) from three independent experiments. Asterisks indicate significant differences, as determined by Student’s *t*-test (*P*<0.05).

To assess the efficiency of VIGS, *CaPAL1* transcript levels were monitored by real-time RT-PCR in empty-vector control (TRV:00) and *CaPAL1*-silenced (TRV:*PAL1*) pepper leaves infected with *Xcv* (Fig. 3). The *CaPAL1* transcript levels decreased significantly in the *CaPAL1*-silenced pepper leaves during virulent or avirulent *Xcv* infection, indicating that *CaPAL1* silencing was effective in suppressing *CaPAL1* expression (Fig. 3).

Real-time RT-PCR analyses were performed to investigate whether silencing of *CaPAL1* altered the expression of defence-related genes in pepper during *Xcv* infection (Fig. 3). Silencing of *CaPAL1* significantly compromised the induction of *CaPAL1* and SA-dependent *CaPR1* (basic PR1) by virulent or avirulent *Xcv* infection. Consequently, the levels of *CaPR1* induced in silenced plants were distinctly lower than those induced in the empty-vector control plants during *Xcv* infection. Silencing of *CaPAL1* significantly inhibited *CaDEF1* (defensin) expression in the healthy pepper leaves. However, the *CaDEF1* levels during infection were similar in both empty-vector control and *CaPAL1*-silenced pepper leaves, although *CaDEF1* was less expressed in silenced leaves 12h after inoculation with the compatible *Xcv* (Fig. 3). Suppression of the induction of the SA-dependent marker gene *CaPR1* by *CaPAL1* silencing was clearly observed among the tested defence genes.

**Fig. 4.**
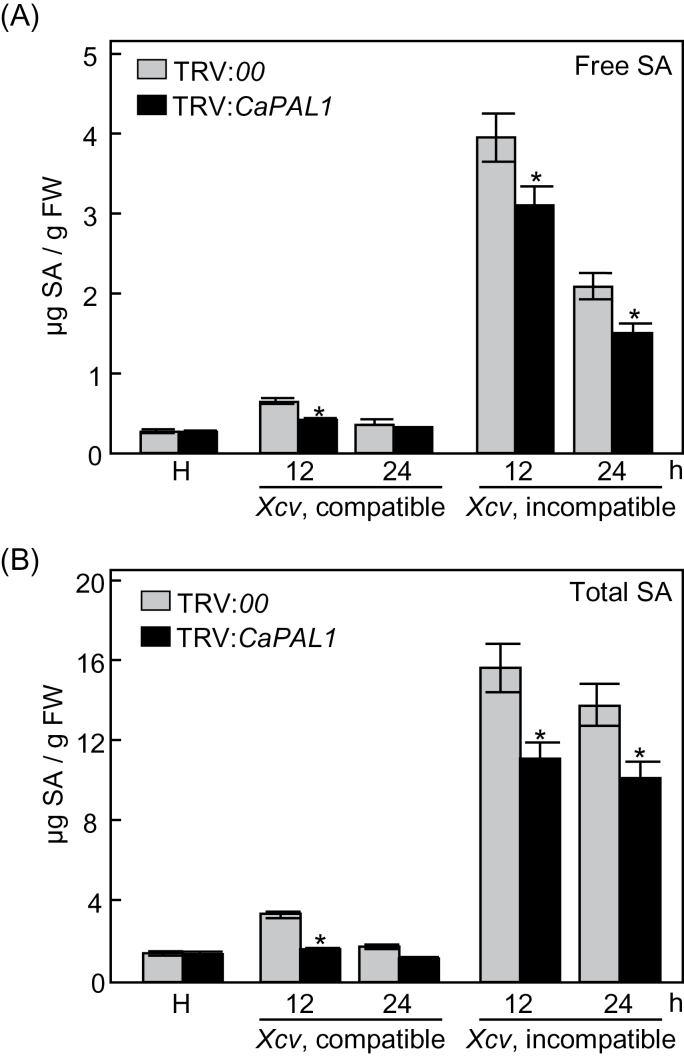
Levels of free (A) and total (B) SA in empty-vector control (TRV:*00*) and *CaPAL1*-silenced (TRV:*CaPAL1*) pepper leaves infected with virulent (compatible) Ds1 and avirulent (incompatible) Bv5-4a strains of *Xcv* (10^7^ cfu ml^–1^). Total SA, free SA plus its glucoside (SAG); H, healthy leaves; FW, fresh weight. Data are the means±SD from three independent experiments. Asterisks indicate significant differences, as determined by Student’s *t*-test (*P*<0.05).

To investigate whether the effect of *CaPAL1* silencing on SA accumulation was related to defence response, we analysed levels of free SA and total SA (free SA plus Glc-conjugated SA) in leaves of the empty-vector and *CaPAL1*-silenced pepper plants infected with compatible Ds1 (virulent) and incompatible Bv5-4a (avirulent) strains of *Xcv* (Fig. 4). *CaPAL1* silencing significantly compromised the induction of SA accumulation during avirulent and virulent *Xcv* infection, although there was no significant difference in the SA induction between the empty-vector and silenced plants 24h after inoculation with the virulent *Xcv*. Taken together, these results indicated that *CaPAL1* and *CaPR1* are involved in SA-dependent defence signalling during *Xcv* infection.

### Increased resistance of *Arabidopsis CaPAL1*-OX transformants to *Pst*

**Fig. 5.**
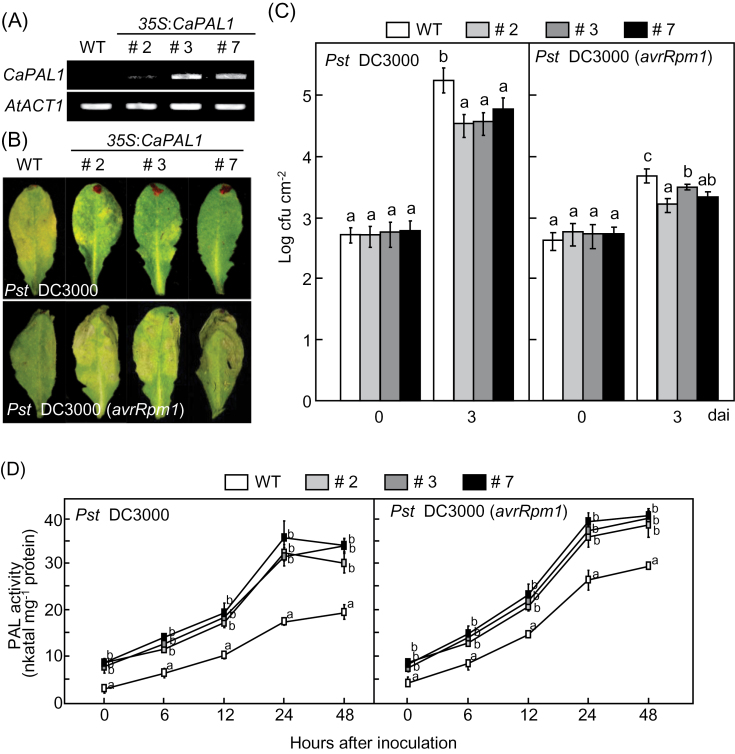
Increased resistance of *CaPAL1*-OX *Arabidopsis* plants to *Pst* DC3000 and DC3000 (*avrRpm1*) infection. (A) RT-PCR analysis of *CaPAL1* expression in leaves of WT and *CaPAL1*-OX transgenic plants. *AtACT1* expression was visualized as a control. (B) Disease symptoms developed on the leaves 2 d after inoculation (10^7^ cfu ml^–1^). (C) Bacterial growth in the leaves of WT and *CaPAL1*-OX transgenic plants 0 and 3 d after inoculation (10^5^ cfu ml^–1^). (D) PAL activity in leaves of WT and *CaPAL1*-OX transgenic plants infected with *Pst* (10^7^ cfu ml^–1^). Data are the means±SD from three independent experiments. Statistical significances according to Fisher’s protected LSD test (*P*<0.05) are indicated by different letters above the data points. (This figure is available in colour at *JXB* online.)

The *Arabidopsis CaPAL1*-overexpression (OX) lines #2, #3, and #7, which constitutively expressed *CaPAL1*, were used to investigate *CaPAL1* gain of function *in planta* (Fig. 5A). We tested whether overexpression of the *CaPAL1* gene in *Arabidopsis* increased the resistance to the hemi-biotrophic bacterium *Pst* DC3000, and DC3000 strains harbouring *avrRpm1*. Severe disease symptoms were observed on the leaves of WT plants but not on *CaPAL1*-OX leaves 2 d after inoculation with virulent *Pst* DC3000 (10^7^ cfu ml^–1^) (Fig. 5B). Severe HR phenotypes against avirulent *Pst* DC3000 (*avrRpm1*) were observed in *CaPAL1*-OX leaves compared with those in WT plants (Fig. 5B). All *CaPAL1*-OX lines exhibited significantly lower bacterial growth compared with that in WT plants 3 d after inoculation with virulent and avirulent *Pst* DC3000 (10^5^ cfu ml^–1^) (Fig. 5C).

We next analysed PAL activity in WT and *CaPAL1*-OX *Arabidopsis* plants during infection with *Pst* DC3000 and *Pst* DC3000 (*avrRpm1*) (Fig. 5D). The levels of PAL activity were slightly higher in healthy leaves of *CaPAL1*-OX plants compared with those in WT plants. As expected, infection with virulent *Pst* DC3000 or avirulent *Pst* DC3000 (*avrRpm1*) induced significantly higher PAL activities in *CaPAL1*-OX transgenic plants compared with the induction of PAL activities in WT plants.

### *CaPAL1* overexpression in *Arabidopsis* stimulates oxidative burst and cell death during *Pst* infection

**Fig. 6.**
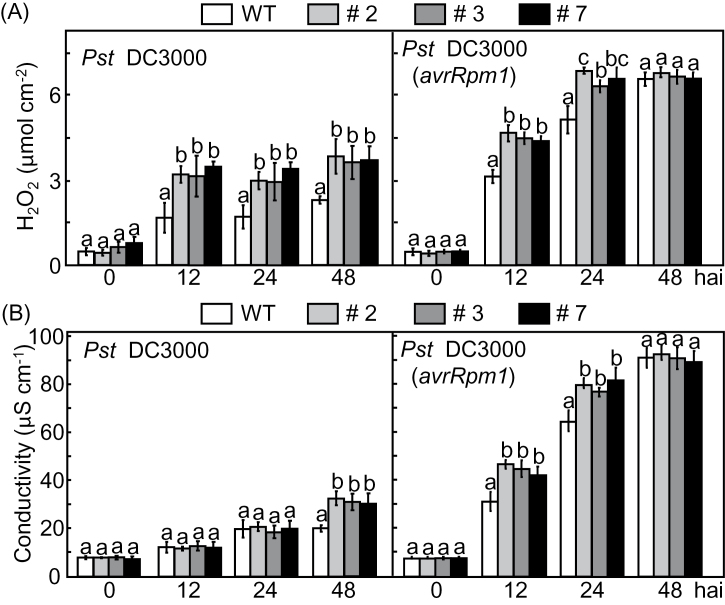
Increased ROS burst and cell death in leaves of *CaPAL1*-OX transgenic *Arabidopsis* plants infected with *Pst* DC3000 and DC3000 (*avrRpm1*). (A) Quantification of H_2_O_2_ from leaf tissues. (B) Quantification of electrolyte leakage from leaf tissues. Statistical significances according to Fisher’s protected LSD test (*P*<0.05) are indicated by different letters above the data points.

We investigated whether *CaPAL1* overexpression altered the oxidative burst and cell death response during *Pst* infection (Fig. 6). H_2_O_2_ accumulation and cell death were quantified using the xylenol orange method and ion conductivity, respectively. H_2_O_2_ accumulation in *CaPAL1*-OX leaves was higher than that in WT leaves 12−48h after inoculation with *Pst* DC3000 and DC3000 (*avrRpm1*) (Fig. 6A). As a cell death indicator, electrolyte leakage from leaf tissues in *CaPAL1*-OX plants was distinctly stimulated by *Pst* infection, compared with that in leaf tissues of WT plants (Fig. 6B). The avirulent DC3000 (*avrRpm1*) infection rapidly and strongly induced ion leakage from *CaPAL1*-OX leaves 12h after bacterial infiltration.

### *CaPAL1* overexpression in *Arabidopsis* stimulates defence-related gene expression and SA accumulation during *Pst* infection

**Fig. 7.**
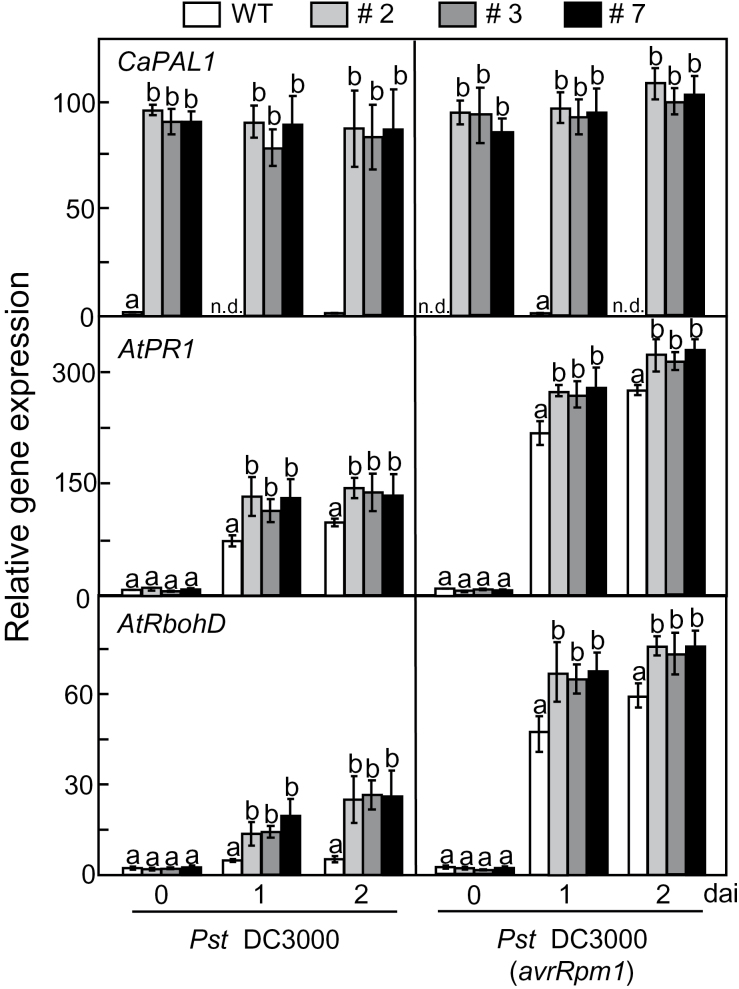
Real-time RT-PCR analyses of the expression of *CaPAL1* and defence-related genes in WT and *CaPAL1*-OX *Arabidopsis* plants infected with *Pst* DC3000 and *Pst* DC3000 (*avrRpm1*). *AtPR1*, pathogenesis-related protein 1; *AtRbohD*, respiratory burst oxidase protein D. *Arabidopsis ACT1* was used as an internal control. The control sample was normalized to 1. Data are the means±SD from three independent experiments. Statistical significances according to Fisher’s protected LSD test (*P*<0.05) are indicated by different letters above the data points.

RT-PCR analyses showed that constitutive *CaPAL1* overexpression was confirmed in leaves of transgenic *Arabidopsis* plants (Fig. 7). Both virulent *Pst* DC3000 and avirulent DC3000 (*avrRpm1*) infections induced significantly higher expression of SA-dependent *AtPR1* and the ROS-producing NADPH oxidase *AtRbohD* in leaves of *CaPAL1*-OX plants than in leaves of WT plants. By contrast, the levels of the oxidative stress-induced marker gene *AtGST1* and the jasmonic acid-dependent marker gene *AtPDF1.2* were similar in WT and *CaPAL1*-OX transgenic leaves (Supplementary Fig. S4 at *JXB* online).

**Fig. 8.**
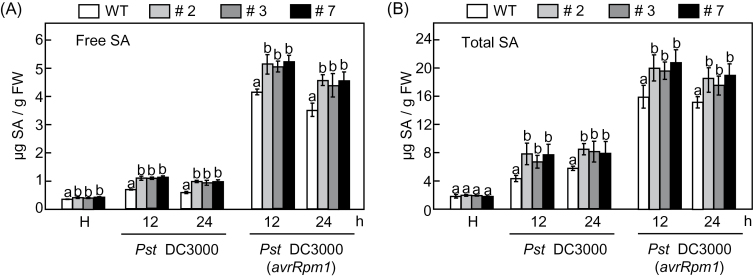
Levels of free (A) and total (B) SA in WT and *CaPAL1*-OX *Arabidopsis* plants infected with virulent *Pst* DC3000 and avirulent *Pst* DC3000 (*avrRPM1*) strains (10^7^ cfu ml^–1^). Total SA, free SA plus its glucoside (SAG); H, healthy leaves; FW, fresh weight. Data are the means±SD from three independent experiments. Statistical significances according to Fisher’s protected LSD test (*P*<0.05) are indicated by different letters above the data points.

We also measured the levels of free SA and total SA (free SA plus Glc-conjugated SA) in leaves of the WT and *CaPAL1*-OX transgenic lines during *Pst* infection (Fig. 8). *CaPAL1* overexpression in *Arabidopsis* significantly induced free SA accumulation in the healthy leaves. Moreover, both virulent *Pst* DC3000 and avirulent *Pst* DC3000 (*avrRpm1*) infections strongly stimulated SA induction in leaves of the *CaPAL1*-OX transgenic lines, compared with that in WT leaves. Notably, the SA induction by avirulent *Pst* DC3000 (*avrRpm1*) infection was much higher than that by virulent *Pst* DC3000 infection. Together, these results suggest that constitutive overexpression of *CaPAL1* in *Arabidopsis* upregulates the SA accumulation during *Pst* infection.

### Increased resistance of *CaPAL1-OX Arabidopsis* to *Hpa* infection

**Fig. 9.**
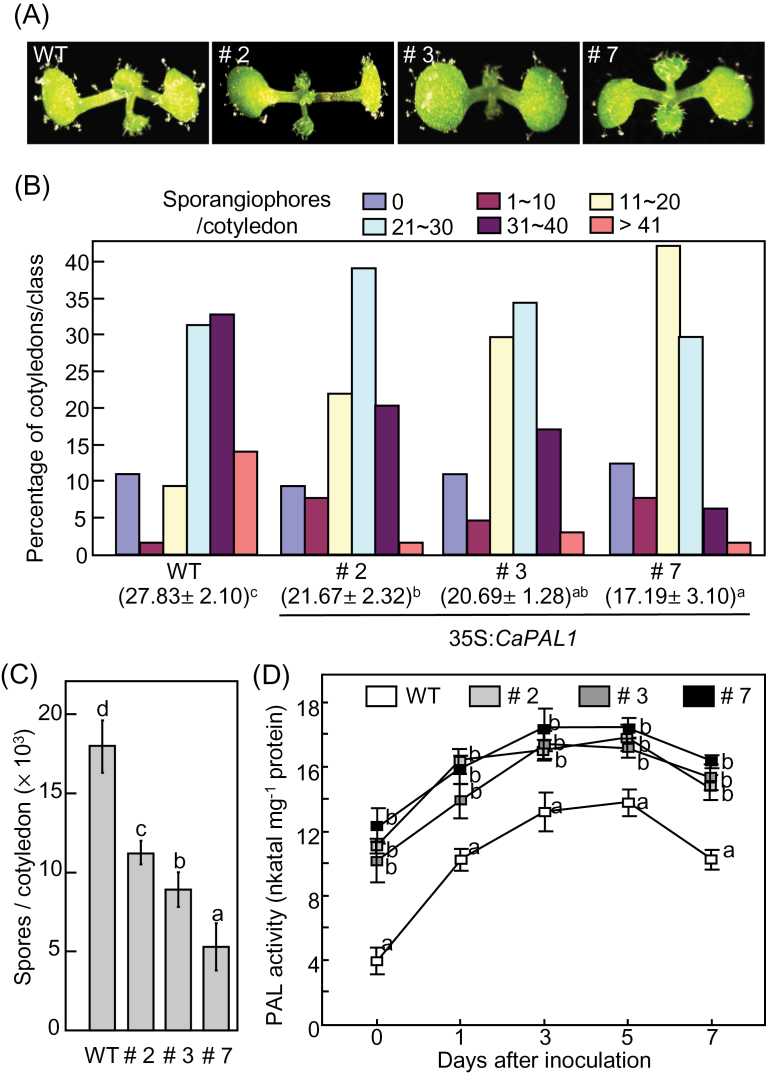
Increased resistance of *Arabidopsis CaPAL1*-OX plants to *Hpa* infection. (A) Disease symptoms on cotyledons of WT and overexpressing transgenic plants 6 d after inoculation with *Hpa* isolate Noco2 (5×10^4^ conidiospores ml^–1^). (B) Quantification of asexual sporangiophores on cotyledons of WT and transgenic plants 6 d after inoculation. The average numbers of sporangiophores±SD are shown below each of the lines tested. (C) Average numbers of spores on cotyledons of WT and transgenic plants. (D) PAL activity in WT and *CaPAL1*-OX transgenic plants infected with *Hpa* Noco2 (5×10^4^ conidiospores ml^–1^). Data in (B) to (D) are the means±SD from three independent experiments. Statistically significant differences between the means were determined by employing the Fisher’s protected LSD test (*P*<0.05). (This figure is available in colour at *JXB* online.)

When inoculated with *Hpa* isolate Noco2 (10^5^ spores ml^–1^), *Hpa* grew well on cotyledons of *Arabidopsis* seedlings (Fig. 9A). Seven days after inoculation, sporangiophores were counted on more than 50 cotyledons (Fig. 9B). Fewer sporangiophores were observed on *CaPAL1*-OX cotyledons compared with those on WT cotyledons (Fig. 9A, B). The infected cotyledons were grouped into five classes based on the number of sporangiophores per cotyledon: 0, 1–10, 11–20, 21–30, 31–40, and >41. The average number of sporangiophores was significantly lower in *CaPAL1*-OX plants than in WT plants (Fig. 9B). *Hpa* produced fewer spores on the cotyledons of *CaPAL1*-OX seedlings than on WT cotyledons (Fig. 9C). We measured PAL activity in WT and *CaPAL1*-OX leaves during *Hpa* Noco2 infection. The induction of PAL activity in *CaPAL1*-OX leaves by *Hpa* Noco2 infection was significantly higher than that in WT leaves (Fig. 9D). Together, these results suggest that *CaPAL1* overexpression confers increased basal resistance to *Hpa* infection in *Arabidopsis* plants.

## Discussion

PALs are encoded by a multi-gene family in plant species such as *Arabidopsis* (Wanner *et al.*, 1995; Cochrane *et al.*, 2004), *Solanum lycopersicum* (Guo and Wang, 2009), *Bambusa oldhamii* (Hsieh *et al*., 2010a, b), and *Phyllostachys edulis* (Gao *et al.*, 2012). PALs are one of the most extensively studied plant enzymes, particularly with respect to their responses to a variety of biotic and abiotic stress (Dixon and Paiva, 1995; MacDonald and D’Cunha, 2007). The biological functions of the *PAL* genes have been investigated through gene silencing in tobacco and disruption of the *PAL1*, *PAL2*, *PAL3*, and *PAL4* genes in *Arabidopsis* (Elkind *et al.*, 1990; Pallas *et al.*, 1996; Rohde *et al.*, 2004; Huang *et al.*, 2010).

In this study, we isolated and functionally characterized the PAL gene (*CaPAL1*) in pepper (*C. annuum*) plants. Based on the observed results, *CaPAL1* was proposed to function as a positive regulator of plant innate immunity. To determine the effect of the loss-of-function or gain-of-function *PAL* gene in pepper plants, we generated *CaPAL1* knockdown pepper plants using the VIGS technique (Liu *et al.*, 2002) and *CaPAL1*-OX transgenic *Arabidopsis* using *Agrobacterium*-mediated transformation (Clough and Bent, 1998). First, the strict conservation of a PAL domain in the deduced amino acid sequence of CaPAL1 suggested that CaPAL1 may function as a PAL enzyme in pepper plants. The suppression or induction of PAL activity in plants during pathogen infection was confirmed by biochemical assays (Song and Wang, 2011) on the *CaPAL1*-silenced pepper plants and the *CaPAL1*-OX transgenic *Arabidopsis* plants. These data suggested that *CaPAL1* expression significantly increases PAL activity, which triggers the immune response in plants.

PAL catalyses the first step of the phenylpropanoid pathway and the synthesis of diverse natural products based on the phenylpropane skeleton, such as hydroxycinnamic acid, stilbenes, and flavonoids, which fulfil many essential roles in higher plants (MacDonald and D’Cunha, 2007). *CaPAL1* transcripts are abundant in root tissues, barely detectable in stem and flower tissues, and not constitutively expressed in leaves, green fruit, and red fruit of healthy pepper plants. Suppression of *PAL* in *Salvia miltiorrhiza* plants results in abnormal phenotypes such as delayed root formation and an underdeveloped root system (Song and Wang, 2011), indicating that PAL is required for root development in plants. Consistent with these results, the constitutive expression of *CaPAL1* in pepper roots supports the hypothesis that PAL activity may be required for proper root formation and development. The upregulation of *CaPAL1* in pepper leaves by avirulent *Xcv* infection suggests that *CaPAL1* plays a crucial role in the induction of plant defence in response to microbial pathogens.

Phenylpropanoid compounds have been proposed to play crucial roles in plant defence to microbial pathogens based on the correlation between rates of phenylpropanoid accumulation and expression of resistance *in vivo* (Dixon and Paiva, 1995; La Camera *et al.*, 2004). *CaPAL1* silencing in pepper plants confers increased susceptibility to *Xcv* infection. Some phenylpropanoid compounds accumulate to high levels in plants that are resistant to an invading pathogen (Dixon, 2001; La Camera *et al.*, 2004). These pathogen-induced phenylpropanoids, such as pterocarpans, isoflavans, stilbenes, and coumarines, act as phytoalexins, which have antimicrobial activity against plant-pathogenic fungi and bacteria (Dixon and Paiva, 1995; Barber *et al.*, 2000; La Camera *et al.*, 2004). The induction of PAL activity by *Xcv* infection was significantly compromised in *CaPAL1*-silenced pepper plants compared with that in empty-vector control plants. The level of PAL activity in *CaPAL1*-silenced plants was lower than that in the empty-vector control plants. These results suggested that PAL activity in pepper plants contributes to the basal and *R* gene-mediated resistance to *Xcv* infection.

SA-dependent signalling controls activation of sophisticated plant defence mechanisms to ward off attacks from microbial pathogens (Delaney *et al.*, 1994; Lu, 2009; Vlot *et al.*, 2009; Robert-Seilaniantz *et al.*, 2011). *CaPAL1* silencing in pepper leaves significantly compromised the induction of SA accumulation and SA-dependent *CaPR1* expression during avirulent and virulent *Xcv* infection. *CaPAL1* overexpression in *Arabidopsis* strongly stimulated SA induction as well as *AtPR1* expression in leaves of the transgenic plants infected with both virulent *Pst* DC3000 and avirulent *Pst* DC3000 (*avrRpm1*). These results suggest that CaPAL1 acts as a positive regulator of SA-dependent defence signalling and downstream defence gene expression. It has been proposed that PAL activity is important for pathogen-induced SA formation in plants (Pallas *et al.*, 1996; La Camera *et al.*, 2004). The levels of free SA produced in pathogen-inoculated leaves of *PAL*-silenced *Nicotiana tabacum* plants were lower than those in the WT control plants (Pallas *et al.*, 1996). The PAL inhibitor 2-aminoindan-2-phosphonic acid abolishes pathogen- or pathogen elicitor-induced SA accumulation in potato, cucumber, and *Arabidopsis* (Meuwly *et al.*, 1995; Mauch-Mani and Slusarenko, 1996; Coquoz *et al.*, 1998). Two pathways of SA biosynthesis have been proposed in plants (Chen *et al.*, 2009). One is the phenylpropanoid pathway controlled by PAL as the first enzyme (Huang *et al.*, 2010), and the other is the chorismate pathway controlled by isochorismate synthase (ICS) (Wildermuth *et al.*, 2001; Catinot *et al.*, 2008; Chen *et al.*, 2009). Previous evidence from *Arabidopsis* has indicated that the chorismate pathway is the most important for SA biosynthesis after stress stimuli (Garcion *et al.*, 2008). In fact, knockout in the two ICS genes (*ics1 ics2* double mutant) in *Arabidopsis* completely abolishes SA accumulation after stress (Garcion *et al.*, 2008). On the other hand, plants knocked out in the four PAL genes from *Arabidopsis* (*pal1 pal2 pal3 pal4* quadruple mutant), which retain 10% of PAL activity, also showed a reduction in the basal levels of SA (up to 25% of WT) and in the pathogen-induced levels (50% of WT) (Huang *et al.*, 2010). However, whether SA is produced via the chorismate pathway in pepper remains to be determined.

In this study, VIGS of *CaPAL1* altered pepper defence-related gene expression. Suppression of the induction of the SA-dependent marker gene *CaPR1* by *CaPAL1* silencing was apparent among the tested defence-related genes, suggesting that *CaPAL1* is required for SA-dependent defence signalling during *Xcv* infection. The oxidative burst and H_2_O_2_ accumulation seem to be essential for the establishment of plant immunity (Alvarez *et al.*, 1998; Apel and Hirt, 2004; Choi *et al.*, 2007). Consistent with these results, VIGS of *CaPAL1* compromised H_2_O_2_ accumulation and cell death during *Xcv* infection, suggesting that *CaPAL1* expression triggers oxidative burst and cell death to promote the plant immune response.

Constitutive overexpression of *CaPAL1* in transgenic *Arabidopsis* lines was used to investigate whether *CaPAL1*-OX affects the defence response and PAL activity when expressed epigenetically. *CaPAL1*-OX plants showed increased resistance to both virulent and avirulent strains of *Pseudomonas syringae* pv. *tomato* (*Pst*) and to virulent *Hpa* Noco2. PAL activity in *CaPAL1*-OX *Arabidopsis* was significantly higher than that in non-inoculated WT plants. PAL activity was increased in both *CaPAL1*-OX and WT plants after *Pst* infection. These results suggested that constitutive expression of *CaPAL1* may lead to the induction of PAL activity in response to *Pst* infection. Although PAL activity in *CaPAL1*-OX transgenic plants was significantly higher than that in WT plants, the levels of induction of PAL activity were similar in *CaPAL1*-OX and WT *Arabidopsis* infected with virulent *Hpa* Noco2. PAL activity in rosette leaves was generally higher than that in cotyledons, suggesting that the phenylpropanoid pathway may be more active in rosette leaves than in cotyledons. During *Pst* infection, the induction of ROS accumulation and ion leakage were higher in *CaPAL1*-OX plants. Expression of the SA-dependent *AtPR1* and the ROS-producing NADPH oxidase *AtRbohD* in leaves of *CaPAL1*-OX plants was higher than that in WT plants. *AtRbohD* was proposed to be required for ROS accumulation in the plant defence response (Torres *et al.*, 2002). Collectively, these results suggest that the *CaPAL* gene is required for the induction of SA-dependent defence signalling events in plants.

## Supplementary data

Supplementary data are available at *JXB* online.

Supplementary Fig. S1. (A) Nucleotide and deduced amino acid sequences of pepper *CaPAL1* cDNA encoding a class family of phenylalanine ammonia-lyase. (B) Comparison of the deduced amino acid sequence of CaPAL1 with phenylalanine ammonia-lyase from other plant species.

Supplementary Fig. S2. Genomic DNA gel blot analysis of *CaPAL1*. Genomic DNA from pepper plants was digested with restriction enzymes *Eco*RI and *Hin*dIII. *CaPAL1* open reading frame cDNA was used as a positive control. Red arrowheads indicate the genomic DNA fragments hybridized with [^32^P]dCTP-labelled *CaPAL1* probe.

Supplementary Fig. S3. RNA gel blot analyses of *CaPAL1* expression in pepper plants at the six-leaf stage. (A) Constitutive expression of *CaPAL1* in various organs of pepper plants. (B) Time course of induction of *CaPAL1* in leaf tissues infected with virulent strain Ds1 and avirulent strain Bv5-4a of *Xanthomonas campestris* pv. *vesicatoria* (10^7^ cfu ml^–1^). The RNA gel blot was hybridized with ^32^P-labelled *CaPAL1* probe.

Supplementary Fig. S4. Real-time RT-PCR analyses of the expression of *AtGST1* and *AtPDF1.2* in WT and *CaPAL1*-OX *Arabidopsis* plants infected with *Pseudomonas syringae* pv. *tomato* (*Pst*) DC3000 and *Pst* DC3000 (*avrRpm1*). *AtGST1*, glutathione *S*-transferase; *AtPDF1.2*, plant defensin 1.2. *Arabidopsis ACT1* was used as an internal control. The control sample was normalized to 1. Data are the means±SD from three independent experiments. Statistical significances according to Fisher’s protected LSD test (*P*<0.05) are indicated by different letters above the data points.

Supplementary Data

## Abbreviations:

DAB: 3,3′-diaminobenzidine
Hpa: *Hyaloperonospora arabidopsidis*
OX: overexpression
PAL: phenylalanine ammonia-lyase
Pst: *Pseudomonas syringae* pv. *tomato*
ROS: reactive oxygen species
SA: salicylic acid
TRV: tobacco rattle virus
VIGS: virus-induced gene silencing
Xcv: *Xanthomonas campestris* pv. *vesicatoria* .
