# Supplementary material for: An important role of the pepper phenylalanine ammonia-lyase gene (PAL1) in salicylic acid-dependent signalling of the defence response to microbial pathogens
Source: J Exp Bot. 2014 Mar 18;65(9):2295–306. doi: 10.1093/jxb/eru109 (PMC4036500; doi:10.1093/jxb/eru109)
Supplement: Supplementary Data [file supp_65_9_2295__index.html]

An important role of the pepper phenylalanine ammonia-lyase gene (PAL1) in salicylic acid-dependent signalling of the defence response to microbial pathogens — An important role of the pepper phenylalanine ammonia-lyase gene (PAL1) in salicylic acid-dependent signalling of the defence response to microbial pathogens — An important role of the pepper phenylalanine ammonia-lyase gene (PAL1) in salicylic acid-dependent signalling of the defence response to microbial pathogens — Supplementary Data 

# An important role of the pepper phenylalanine ammonia-lyase gene (*PAL1*) in salicylic acid-dependent signalling of the defence response to microbial pathogens

## Supplementary Data

Data files

**Files in this Data Supplement:**

- Supplementary Data - Supplementary Data
