# Supplementary material for: An important role of the pepper phenylalanine ammonia-lyase gene (PAL1) in salicylic acid-dependent signalling of the defence response to microbial pathogens
Source: J Exp Bot. 2014 Mar 18;65(9):2295–306. doi: 10.1093/jxb/eru109 (PMC4036500; doi:10.1093/jxb/eru109)
Supplement: Supplementary Data [file supp_eru109_jexbot107995_file001.pdf]

An important role of the pepper phenylalanine ammonia-lyase gene (*CaPAL1*) for salicylic acid-dependent signalling of defence response to microbial pathogens .

Dae Sung Kim and Byung Kook Hwang

SUPPLEMENTARY DATA

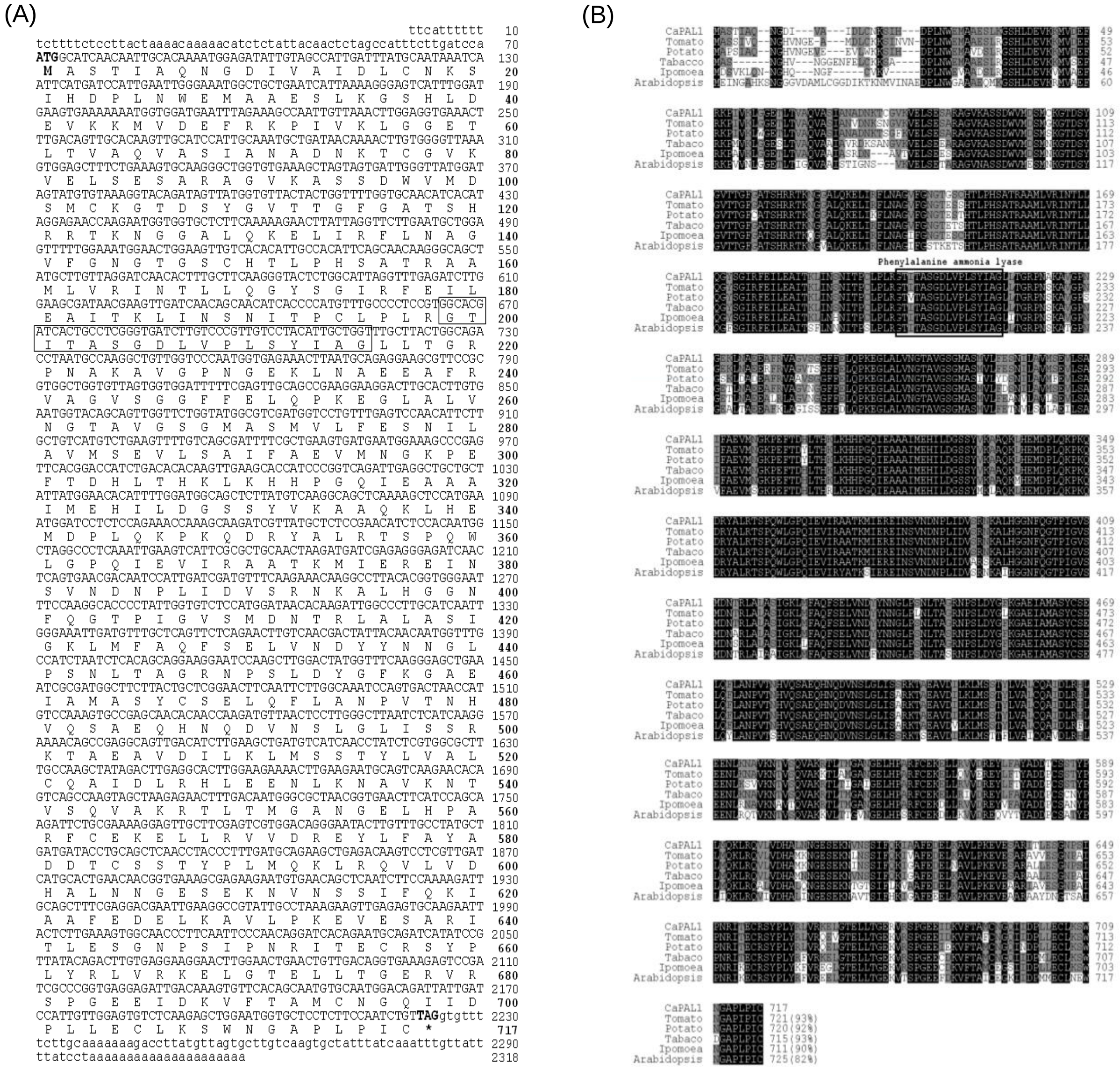

Supplementary Fig. S1. Kim and Hwang

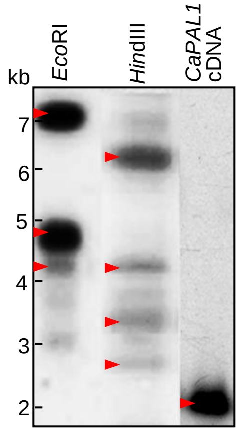

Supplemtary Fig. S2. Kim and Hwang

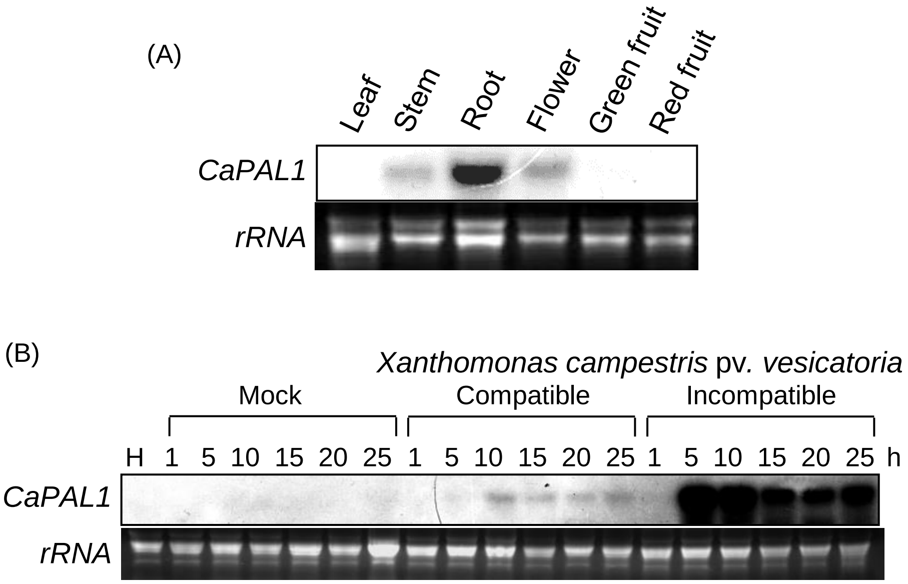

Supplementary Fig. S3. Kim and Hwang

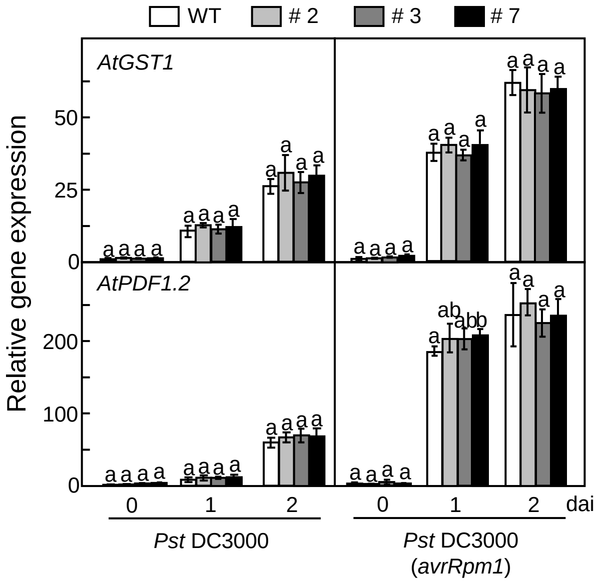

Supplementary Fig. S4. Kim and Hwang
